# Efficacy and safety of the subcutaneous implantable cardioverter defibrillator in patients with and without obesity: An international, bicentric retrospective registry

**DOI:** 10.1007/s00392-025-02834-x

**Published:** 2025-12-17

**Authors:** Christian Gold, Flora Diana Gausz, Anna Vagvolgyi, Florian Hecker, Jana Kupusovic, Marton Miklos, Tamas Szili-Torok, David M. Leistner, Reza Wakili, Julia W. Erath, Mate Vamos

**Affiliations:** 1 Department of Cardiology, Goethe University Hospital, Theodor-Stern-Kai 7, 60590 Frankfurt am Main, Germany; 2 Cardiology Center, Department of Internal Medicine, University of Szeged, Szeged, Hungary; 3 Endocrinology and Diabetology Center, Department of Internal Medicine, University of Szeged, Szeged, Hungary; 4 Department of Cardiac Surgery, Goethe University Hospital, Frankfurt am Main, Germany

**Keywords:** S-ICD, Obesity, DFT, Device-related complications, Device therapy

## Abstract

**Aim:**

This study evaluated the efficacy and safety of the subcutaneous implantable cardioverter defibrillator (S-ICD) in patients with obesity.

**Methods:**

In this bicentric, retrospective study, S-ICD recipients were divided into two groups based on body mass index (BMI: < 30 kg/m^2^ and ≥ 30 kg/m^2^). Defibrillation testing (DFT) failure, shock impedance, rates of appropriate and inappropriate shock, long-term complications, survival, and device-related or cardiac rehospitalizations were compared.

**Results:**

Of the 120 patients included, most baseline characteristics were similar between patients with (*n* = 30) and without obesity (*n* = 90), except for a higher prevalence of diabetes in the group with obesity. The first shock during DFT was similarly effective (99 vs. 100%), although, shock impedance was significantly higher in patients with obesity (59 vs. 74 Ω; *p* = 0.011). There was no difference between the groups regarding the incidence of appropriate (hazard ratio [HR] 0.70, 95% confidence interval [CI] 0.21–2.34, *p* = 0.584), and inappropriate shocks (HR 0.92, 95% CI 0.23–3.48, *p* = 0.902). Non-infectious complications occurred numerically more often in obese patients (16.7% vs. 4.9%, *p* = 0.058), while device-associated infections were more frequent among non-obese patients (7.4% vs. 0%, *p* = 0.188). The risk of all-cause mortality (HR 0.32; 95% CI 0.11–0.97; *p* = 0.141), device-related (HR 0.52; 95% CI 0.14–1.90; *p* = 0.395) or cardiac rehospitalization (HR 1.06; 95% CI 0.48–2.32; *p* = 0.890) were similar between the groups.

**Conclusion:**

Despite higher shock impedance during DFT at S-ICD implantation, shock efficacy remained stable during implantation and follow-up in both groups. Fewer infectious but more system-specific complications may manifest in patients with obesity compared to non-obese patients.

**Graphical Abstract:**

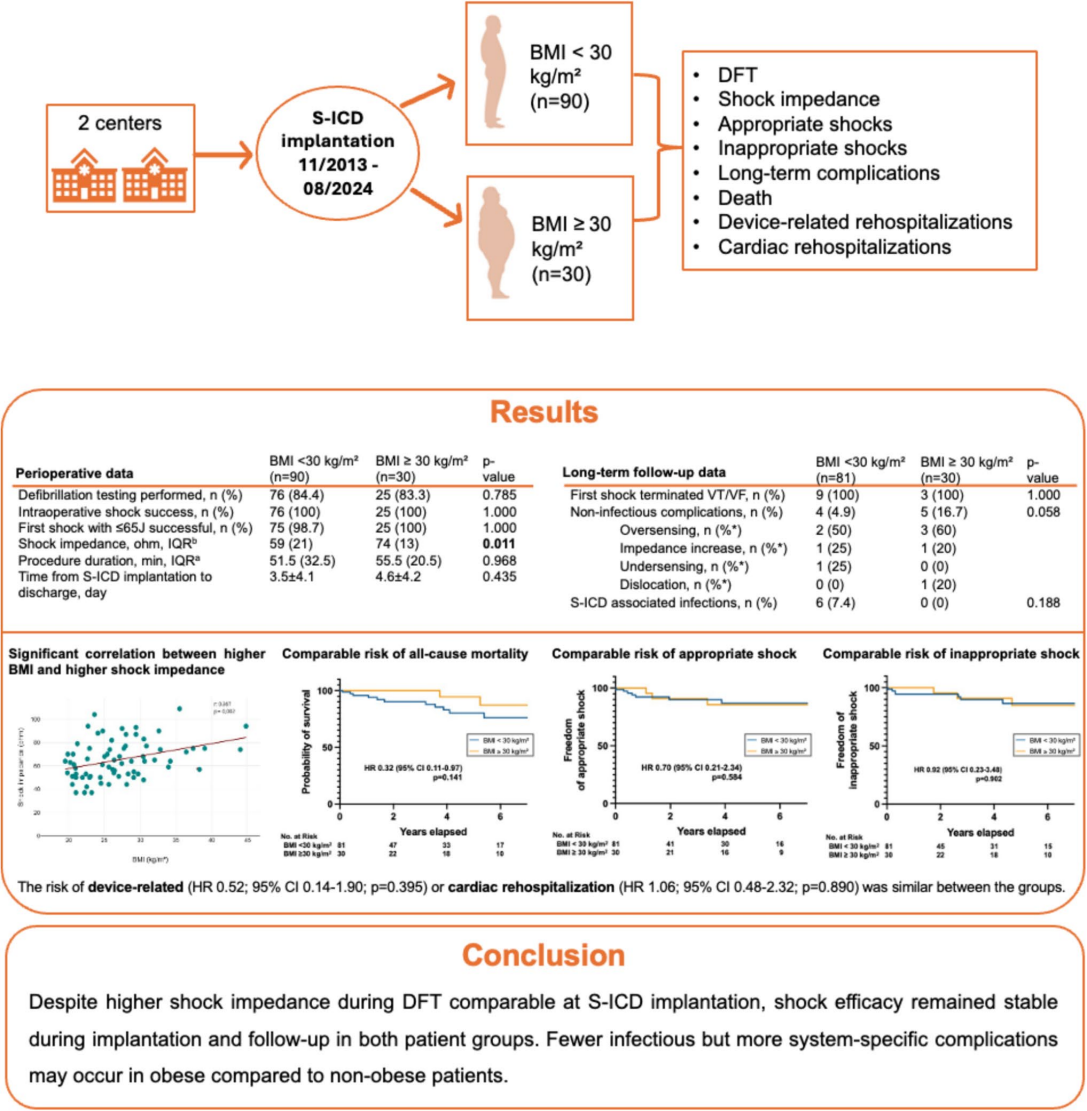

## Introduction

Sudden cardiac death (SCD) is still one of the main reasons for cardiovascular death. To prevent SCD, the implantation of a defibrillator (ICD) has proven to significantly reduce mortality in well-selected patients [1]. The subcutaneous implantable cardioverter-defibrillator (S-ICD) has emerged as a safe and effective alternative to transvenous systems for the prevention of SCD, especially in patients at high risk of lead-related complications or endocarditis [2–9].

Obesity, defined by the World Health Organization (WHO) (10) as a body mass index (BMI) of ≥ 30 kg/m^2^, is a progressive, chronic condition, whose global prevalence has risen dramatically in recent decades, contributing to numerous comorbidities. These include cardiovascular disease, type 2 diabetes mellitus, and chronic kidney disease, which are now increasingly recognized as interconnected components of the cardiovascular-kidney-metabolic (CKM) syndrome [11–15]. Importantly, obesity has also been associated with abnormal ventricular repolarization and an increased risk of ventricular arrhythmias and sudden cardiac death, particularly in individuals with underlying cardiac diseases [16, 17] Consequently, the number of individuals with obesity receiving ICDs—including S-ICDs—is expected to rise in the coming decades.

Obesity-related anatomical factors may affect the eligibility (e. g. pre-implantation electrocardiographic [ECG] screening), efficacy, and safety of S-ICD therapy. For instance, there are well-known procedural challenges that may arise in case of severe obesity, where the device's extravascular configuration and deeper subcutaneous positioning beneath thick adipose layers may hinder optimal lead fixation and increase the risk of dislodgement, suboptimal vector orientation, or elevated defibrillation threshold [18, 19]. Higher BMI and the presence of adipose tissue between the lead coil and sternum or between the generator and skin are critical elements of the PRAETORIAN score, which predicts defibrillation success and guides implant positioning [20, 21]. Some studies have also shown that patients with obesity, undergoing S-ICD implantation, may experience higher complication rates [5]. This has led to hesitation in using S-ICD in this population, as demonstrated by data from European Heart Rhythm Association (EHRA) prospective survey showing a lower prevalence of overweight and obesity among S-ICD recipients compared to those receiving transvenous ICDs [22].

A recent meta-analysis systematically evaluated the efficacy and safety of S-ICDs in patients with obesity [23] including 29 studies and a total of 20,486 patients, comparing outcomes in individuals with versus without obesity. Regarding some of the above-described concerns, the analysis provided reassuring findings. No statistically significant difference was found in baseline BMI between patients who passed or failed the preimplantation ECG screening. In addition, inappropriate shock rates did not differ significantly between the different body weight groups. However, higher BMI was associated with elevated defibrillation thresholds during defibrillation testing (DFT), increased likelihood of lead malposition or suboptimal device positioning, higher complication rates, and a non-significant trend toward more appropriate shocks. Importantly, for certain endpoints the available evidence was based on a limited number of publications, and several key long-term outcomes, including survival and rehospitalization rates, which could not be systematically assessed due to insufficient published data.

Therefore, the aim of our study was to address these evidence gaps by providing real-world, long-term data from an all-comer, international cohort of S-ICD recipients.

## Methods

### Patient population

This was a bicentric, retrospective, observational study that included consecutive patients undergoing S-ICD (Boston Scientific, St. Paul, MN, USA) implantation between November 2013 and August 2024 in two European referral cardiology centers (University Hospital Frankfurt, Germany; University of Szeged, Hungary). All patients were over 18 years old and had met standard ICD indications. The decision to implant an S-ICD was made in accordance with current guideline recommendations. In patients without indication for bradycardia pacing, cardiac resynchronization therapy, or anti-tachycardia pacing as a class IIa indication [1], and in those for whom transvenous lead placement was considered undesirable (e.g., younger age, prior infection, limited vascular access or prior lead failure) as a class I indication [24]. In all candidates, pre-implant S-ICD ECG screening using the manufacturer’s template was mandatory, and only those who successfully passed the screening were considered eligible for implantation.

The patient cohort was divided into two groups according to their respective BMI, following the WHO’s definition [10], into patients with obesity (≥ 30 kg/m^2^) and without obesity (< 30 kg/m^2^).

The study was approved by the institutional review boards of the participating centers, and it conforms to the ethical guidelines of the Declaration of Helsinki.

### Periprocedural workflow

Procedures were performed by experienced operators applying either local anesthesia, conscious sedation, or general anesthesia, according to the center’s standards at the time of the implantation. All patients received a single-dose antibiotic prophylaxis according to the standard operating procedure of the implanting center. As implantation technique, we preferably applied the two-incision technique as the standard implantation technique in both centers in accordance with the manufacturer´s recommendations [25]. At the end of the implantation, acute defibrillation testing (DFT) was routinely performed, except in cases where the implanting physician decided to omit it for any reason. Ventricular fibrillation (VF) was induced with 50-Hz transthoracic pacing. Detection was performed automatically by the device, after which an initial 65-J shock was delivered. In a few exceptional cases, DFT was performed with an initial shock lower or higher than this, based on the operator's decision. If the first 65 J shock proved to be ineffective, the DFT was repeated with an 80 J shock, using alternating polarity. After implantation, all shocks were programmed to 80 J maximum energy.

### Data collection

Data was retrospectively collected from the index hospitalization at the time of initial S-ICD implantation, 6 weeks after the implantation, and at each follow-up visit, which took place every six months or at the time of unscheduled visits in the out- or in-patient clinic. Data collection included patient characteristics, such as age, indication for S-ICD implantation, left ventricular ejection fraction (LVEF), BMI, and relevant comorbid conditions. The Charlson Comorbidity Index (CCI) [26], as a validated assessment score designed specifically to predict long-term mortality in the presence of a range of comorbid conditions, was calculated at baseline for every patient. LVEF and left ventricular end-diastolic diameter (LVEDD) were evaluated by experienced cardiologists independently, as well as by automatic measurements (GE Healthcare, AutoEF, Chicago, IL, USA) within 4 weeks of device implantation. Pertinent medication use (beta-blockers, ACE-inhibitors or angiotensin receptor blockers, aldosterone antagonists, angiotensin receptor neprilysin inhibitors, class III antiarrhythmics, anticoagulants) was documented. Further, laboratory results, such as creatine, C-reactive protein, and white blood cell count, were additionally collected.

All relevant information was entered into a customized database. For missing data, particularly in the case of missed follow-up visits, family members, treating physicians, or other hospitals were contacted to retrieve the missing information.

### Study endpoints

First, implantation-related outcomes, such as failure to terminate VF during perioperative defibrillation testing with ≤ 65 J or with maximal energy, and shock impedance during DFT were evaluated. Moreover, the PRAETORIAN score [20, 21], procedure duration, and the time from S-ICD implantation to discharge were collected and compared between the two patient groups.

As long-term outcomes, time to first appropriate and inappropriate shocks, clinical shock efficacy, complication rate, time to all-cause mortality, and the risk of device-related or cardiac rehospitalizations were evaluated and compared between the two patient groups. System-related complications were grouped as either infectious or non-infectious.

In addition, a subgroup analysis was performed including only patients implanted for primary prevention to evaluate potential differences in long-term outcomes between BMI groups.

### Statistics

Statistical analysis was performed using the SPSS version 22 software (IBM, Chicago, IL, USA). Categorical variables are presented as numbers and percentages, and continuous variables are summarized as means and standard deviations (SD), or median and interquartile ranges (IQR), depending on the normality of distribution assessed by the Shapiro–Wilk test.

Baseline, perioperative, and follow-up characteristics were compared using the Mann–Whitney U-test and the two-sample t-test (continuous variables), and the Chi-square test or the Fisher exact test (categorical variables). For correlation, the Spearman-Rho correlation method was used for non-parametrically distributed variables. Binary logistic regression was used to evaluate associations between categorical outcomes and covariates. Survival analysis was performed using Kaplan–Meier analysis. Survival curves were compared using the log-rank test and Cox regression analysis, along with hazard ratios (HRs) and 95% confidence intervals (CIs). Only two-sided tests were used, and *p*-values < 0.05 were considered statistically significant.

## Results

### Baseline characteristics

A total of 123 patients who underwent S-ICD implantation were identified. Three patients were excluded due to missing height and weight data, which made the BMI determination impossible. Therefore, the final analysis included 120 patients, of whom 90 had a BMI < 30 kg/m^2^ and 30 had a BMI ≥ 30 kg/m^2^.

The study included predominantly male patients in both groups (75.6 vs. 70.0% for BMI < 30 kg/m^2^ and BMI ≥ 30 kg/m^2^). The mean age of patients in both groups was about 50 years. Patients with obesity (BMI: 35.0 ± 4.1 kg/m^2^) more often had diabetes at implantation (14.4% vs. 31.0%, p = 0.045) compared to patients without obesity (BMI 24.3 ± 2.9 kg/m^2^). There were no differences between the two groups regarding mean LVEF, presence of atrial fibrillation, and the prevalence of ischemic heart disease at the time of implantation. Patients with obesity tended to have S-ICD implanted for primary prevention more often than patients without obesity, without reaching statistical significance (69% vs. 48.9%, p = 0.059). The CCI, as a predictor of ten-year mortality, was also comparable between both groups (77% vs. 83.5%, p = 0.704). Baseline heart failure medication, group III antiarrhythmic therapy, as well as anticoagulation therapy, were well balanced between the groups. The baseline characteristics of the cohorts are listed in detail in Table 1.

**Table 1:** Baseline patient characteristics

	BMI < 30 kg/m^2^ (*n* = 90)	BMI ≥ 30 kg/m^2^ (*n* = 30)	*p*-value
Male sex, n (%)	68 (75.6)	21 (70.0)	0.547
Age at implantation, years	49.7 ± 16	50.6 ± 16.2	0.787
BMI, kg/m^2^	24.3 ± 2.9	35.0 ± 4.1	** < 0.001**
Height, cm	177 ± 9.1	175 ± 11.3	0.226
Weight, kg	76.7 ± 12.5	108 ± 21.4	** < 0.001**
Atrial fibrillation, n (%*)^a^	21 (23.6)	8 (26.7)	0.712
LVEF, %^b^	37.3 ± 15.2	36.7 ± 14.4	0.856
LVEDD, mm^c^	56.8 ± 10.3	60.2 ± 10.0	0.146
CKD, n (%)^d^	18 (20.0)	4 (13.8)	0.587
ICM, n (%)^a^	37 (41.1)	11 (36.7)	0.636
PAD, n (%)^d^	6 (6.7)	1 (3.4)	1.000
Diabetes, n (%)^d^	13 (14.4)	9 (31.0)	**0.045**
History of stroke, n (%)^d^	7 (7.8)	2 (6.9)	1.000
History of MI, n (%)^a^	31 (34.8)	9 (30.0)	0.628
Primary prevention, n (%)^d^	44 (48.9)	20 (69.0)	0.059
Charlson Comorbidity Index, %, IQR^e^	77 (51)	83.5 (61)	0.704
Baseline medication			
Class III AAD, n (%)^a^	7 (7.9)	5 (16.7)	0.175
Beta-blocker, n (%)^a^	81 (91.0)	30 (100)	0.089
MRA, n (%)^a^	49 (55.1)	18 (60.0)	0.637
ACE inhibitor or ARB^a^	43 (48.3)	19 (63.3)	0.154
ARNI, n (%)^a^	22 (24.7)	7 (23.3)	0.879
DOAK or VKA^e^	29 (33.0)	15 (50.0)	0.095

a) Missing for 1 patient without obesity; b) Missing for 7 patients without obesity and 2 patients with obesity; c) Missing for 12 patients without obesity and 3 patients with obesity; d) Missing for 1 patient with obesity; e) Missing for 2 patients without obesity

*AAD* antiarrhythmic drugs, *ACE* Angiotensin-converting enzyme, *ARB* angiotensin receptor blockers, *ARNI* angiotensin receptor neprilysin inhibitor, *BMI* body mass index, *CKD* chronic kidney disease, *DOAK* Direct oral anticoagulants, *ICM* ischemic cardiomyopathy, *IQR* Interquartile range, *LVEF* left ventricular ejection fraction, *LVEDD* left ventricular end-diastolic diameter, *MI* myocardial infarction, *MRA* mineralocorticoid receptor antagonist, *n* number, *PAD* peripheral artery disease, *VKA* Vitamin K antagonists

### Implantation-related outcomes

**Fig. 1:**
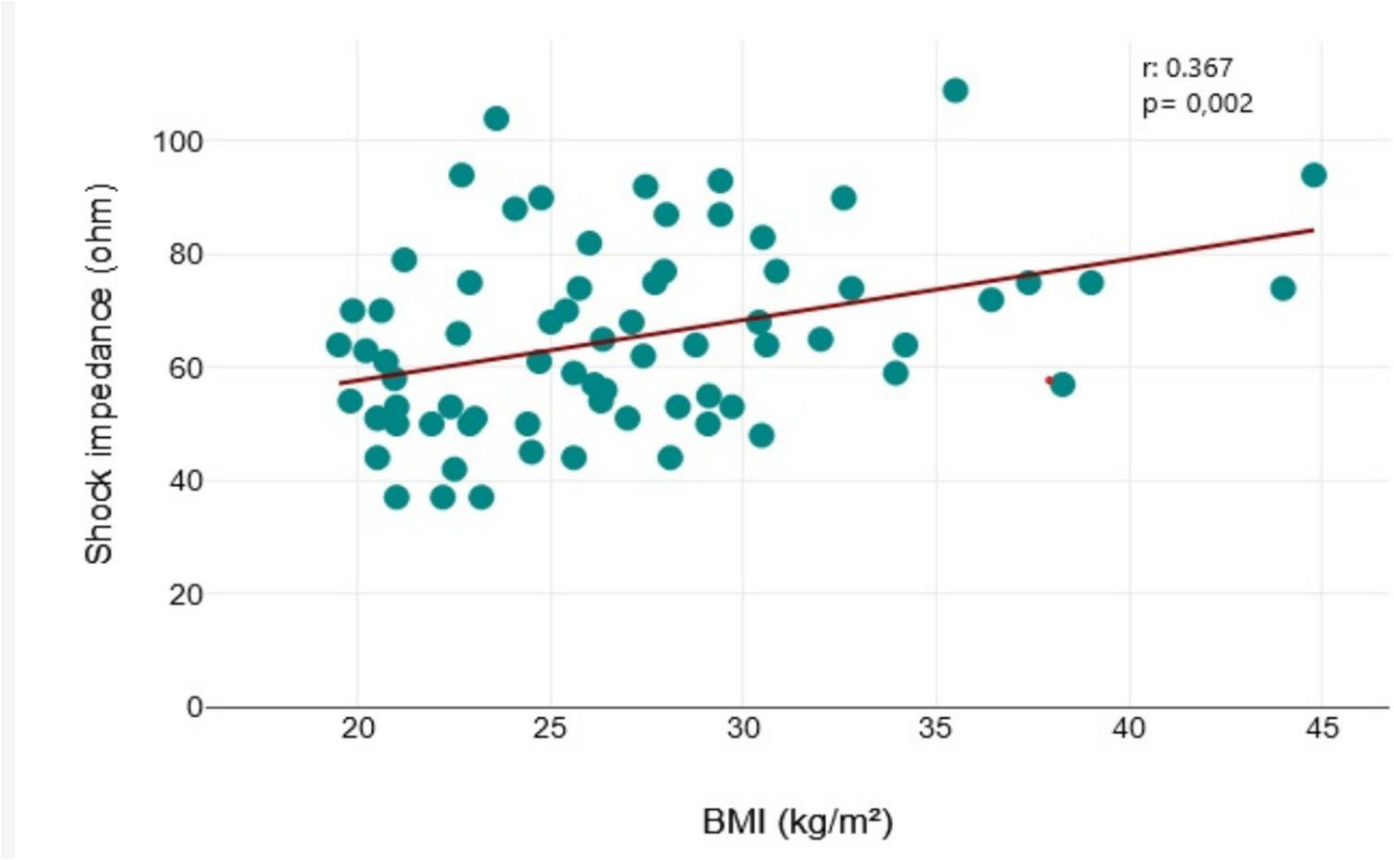
Correlation between shock impedance and BMI at the time of DFT. The scatterplot showing a significant correlation between higher BMI and higher shock impedance. BMI: body mass index; DFT: defibrillation testing

Perioperative data is presented in Table 2. Seventy-six patients without obesity (84.4%) and twenty-five patients with obesity (83.3%) underwent DFT at implantation. Intraoperative testing demonstrated 100% shock success in both BMI groups, with the first shock being effective in nearly all cases. Specifically, the first shock with ≤ 65 J was successful in 98.7% of patients with BMI < 30 kg/m^2^ and in 100% of those with BMI ≥ 30 kg/m^2^. Shock impedance was significantly higher in the obesity group compared to patients without obesity (74 vs. 59 Ω; *p* = 0.011) also yielding a significant correlation between higher BMI and higher shock impedance (r = 0.37, *p* < 0.001) (Fig. 1). The PRAETORIAN score was available in 31 patients (26 without and 5 with obesity). The median score was higher in patients with obesity (150 [90]) compared with those without obesity (30 [30]) (*p* = 0.038). The reasons for not performing DFT testing were predominantly either the presence or suspicion of intracardiac thrombi, or repetitive spontaneous conversion of VF in sinus rhythm (SR) after initial induction.

**Table 2:** Perioperative data

	BMI < 30 kg/m^2^ (*n* = 90)	BMI ≥ 30 kg/m^2^ (*n* = 30)	*p*-value
DFT performed, n (%)	76 (84.4)	25 (83.3)	0.785
Intraoperative shock success, n (%)	76 (100)	25 (100)	1.000
First shock with ≤ 65 J successful, n (%)	75 (98.7)	25 (100)	1.000
Shock impedance, ohm, IQR^a^	59 (21)	74 (13)	**0.011**
Procedure duration, min, IQR^b^	51.5 (32.5)	55.5 (20.5)	0.968
PRAETORIAN Score, IQR^c^	30 (30)	150 (90)	**0.038**
Time from S-ICD implantation to discharge, day	3.5 ± 4.1	4.6 ± 4.2	0.435

a) Missing for 35 patients without obesity and 13 patients with obesity; b) Missing for 58 patients without obesity and 18 patients with obesityc) Missing for 64 patients without obesity and 25 patients with obesity

*DFT* defibrillation threshold testing, *IQR* interquartile range

The median duration of S-ICD implantation was comparable between the two cohorts (51.5 vs. 55.5 min, *p* = 0.968). The total duration of hospitalization from S-ICD implantation to discharge was also comparable in both groups (3.5 ± 4.1 vs 4.6 ± 4.2 days for patients without and with obesity, *p* = 0.435).

### Long-term outcomes

**Fig. 2:**
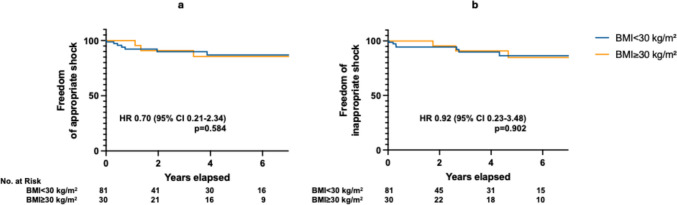
Appropriate **a**) and inappropriate **b**) S-ICD shocks during follow-up. BMI: body mass index; CI: confidence interval; HR: hazard ratio; Nr: number; S-ICD: subcutaneous implantable cardioverter defibrillator

The mean follow-up time was 42 ± 35 months, based on follow-up data available for 111 patients (81 with BMI < 30 kg/m^2^ and 30 with BMI ≥ 30 kg/m^2^). There was no significant difference between the two groups regarding the risk of appropriate shocks (HR 0.70, 95% CI 0.21–2.34, *p* = 0.584) (Fig. 2A), and the risk of inappropriate shocks (HR 0.92, 95% CI 0.23–3.48, *p* = 0.902) (Fig. 2B). The success rate of the first clinical shock in terminating ventricular tachycardia (VT) or ventricular fibrillation (VF) was 100% in both the BMI < 30 kg/m^2^ (9/9) and BMI ≥ 30 kg/m^2^ (3/3) groups (Table 3).

**Table 3:** Long-term follow-up data

	BMI < 30 kg/m^2^ (*n* = 81)	BMI ≥ 30 kg/m^2^ (*n* = 30)	*p*-value
First shock terminated VT/VF, n (%)	9 (100)	3 (100)	1.000
Non-infectious complications, n (%)	4 (4.9)	5 (16.7)	**0.058**
Oversensing, n (%*)	*2 (50)*	*3 (60)*	
Impedance increase, n (%*)	*1 (25)*	*1 (20)*	
Undersensing, n (%*)	*1 (25)*	*0 (0)*	
Dislocation, n (%*)	*0 (0)*	*1 (20)*	
S-ICD associated infections, n (%)	6 (7.4)	0 (0)	0.188
Lead -infection n (%*)	*1 (16.7)*	*-*	
Pocket infection n (%*)	*5 (83.3)*	*-*	

^*^% of total complications within group

*BMI* body mass index, *DFT* defibrillation testing, *S-ICD* subcutaneous implantable cardioverter defibrillator, *VT* ventricular tachycardia, *VF* ventricular fibrillation

A numerically higher number of non-infectious complications was observed in the patients with obesity group, without reaching statistical significance (16.7% vs. 4.9% for patients with and without obesity, *p* = 0.058) (Table 3). The leading cause of non-infectious complications was oversensing, occurring in five out of a total of nine cases, followed by an increase of impedance in two patients and one case of undersensing, and one case of lead dislocation. All documented non-infectious complications required an intervention. Device-related infections were only recorded in patients without obesity, with a total of six documented cases, one lead infection and 5 pocket infections. In a univariate logistic regression analysis, diabetes was not significantly associated with the occurrence of infections (OR 0.94, 95% CI 0.10–8.58, *p* = 0.96).

**Fig. 3:**
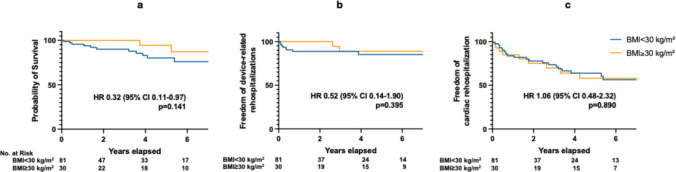
Long-term survival (**a**), risk of device-related (**b**) and cardiac rehospitalizations (**c**). BMI: body mass index; CI: confidence interval; HR: hazard ratio

All-cause death occurred in 14 patients, of whom 12 were patients without obesity, and two patients with obesity, without reaching statistical significance (HR 0.32, 95% CI 0.11–0.97, *p* = 0.141) (Fig. 3a).

There was no difference between patients without or with obesity in rates of device-related hospitalizations (HR 0.52, 95% CI 0.14–1.90, *p* = 0.395) (Fig. 3b) or cardiac rehospitalizations (HR 1.06, 95% CI 0.48–2.32, *p* = 0.890) (Fig. 3c).

As the proportion of primary-prevention patients differed slightly between BMI groups (69.0% for obese vs. 48.9% for non-obese patients, *p* = 0.059), a subgroup analysis was performed including only patients implanted for primary prevention. Notably, there were no significant differences between obese vs non-obese BMI patient groups regarding long-term outcomes: hazard ratios were 1.69 (95% CI 0.11–27.03, *p* = 0.711) for appropriate and 1.18 (95% CI 0.26–5.30, *p* = 0.827) for inappropriate shocks. Non-infectious complications occurred more frequently in patients with obesity (OR 4.63, 95% CI 0.77–27.68, *p* = 0.095), whereas infectious complications were only observed in the non-obesity group (*n* = 2, *p* = 0.554). The hazard ratio for all-cause death was 0.78 (95% CI 0.14–4.29, *p* = 0.779), for device-related rehospitalization 0.32 (95% CI 0.04–2.74, *p* = 0.297), and for cardiac rehospitalization 2.62 (95% CI 0.59–11.70, *p* = 0.208).

## Discussion

### Main findings

The current bicentric, observational study evaluated whether obesity (i.e. BMI ≥ 30 kg/m^2^) affects the efficacy and safety of S-ICD therapy, with particular attention to defibrillation performance and long-term outcomes—an increasingly relevant clinical question given the rising prevalence of obesity in ICD-eligible populations. Despite the significantly higher shock impedance values observed during intraoperative DFT in patients with obesity, the immediate shock effectiveness remained excellent and comparable between patients with and without obesity. Similarly, the clinical efficacy of S-ICD therapy during follow-up, as reflected by the success of the first therapeutic shock in terminating ventricular arrhythmias, was 100% in both BMI groups. No significant differences were observed in the incidence of either appropriate or inappropriate shocks during long-term follow-up. Although non-infectious device-related complications occurred more frequently in patients with obesity, device-related infections were observed only in patients without obesity. Furthermore, there were no significant differences in all-cause mortality, device-related rehospitalizations, or cardiac rehospitalizations between patients with and without obesity.

Taken together, our results indicate that S-ICD implantation is equally effective and safe in patients with and without obesity regarding both periprocedural performance and long-term clinical outcomes. Importantly, while prior literature has primarily focused on implantation-related parameters or complication rates, data on long-term survival and follow-up outcomes in S-ICD recipients with obesity remain limited. Our study addresses this gap by providing real-world, long-term evidence from an all-comer, international cohort, thereby contributing relevant and clinically meaningful data to support the broader applicability of S-ICD therapy in this growing patient population. These data also highlight potentially distinct complication patterns in patients with obesity compared to their counterparts without obesity.

### Implantation-related outcomes

The overall success rate of defibrillation testing was similar between patients with and without obesity in the current study, and more importantly, was 100% in patients with obesity at any delivered energy. Although a meta-analysis of various studies on DFT reported a higher rate of unsuccessful or elevated defibrillation threshold in patients with obesity, and demonstrated that BMI—evaluated as a continuous variable (per 1 kg/m^2^ increase)—was associated with increased DFT [23], the clinical relevance of this association appears to be limited. This is further supported by the fact that in real-world clinical scenarios, the system delivers 80-J shocks for ventricular arrhythmias—substantially higher than the energy levels commonly used during DFT testing. For instance, in the study by Biffi et al., where testing started with lower energies as the standard 65 J, a difference in DFT thresholds between individuals with and without obesity was observed only at 40 J [27]. These findings are in line with our observation that the S-ICD achieved 100% success also in terminating clinical arrhythmias regardless of BMI category.

The shock impedance, as well as the PRAETORIAN score, were significantly higher in patients with obesity in our cohort. This is consistent with the findings of the aforementioned meta-analysis, which also reported elevated shock impedance values in individuals with obesity during device implantation [23]. Nevertheless, despite the correlation between higher BMI and increased impedance as well as higher PRAETORIAN score, this did not translate into reduced defibrillation efficacy at therapeutic energy levels. PRAETORIAN data, however, was available for only a very limited number of patients and should therefore be interpreted with caution. In addition, larger observational studies have demonstrated high DFT success rates in large patient cohorts (up to 99% in 4900 patients) [28]. The smaller number of patients who underwent DFT testing in our study likely limited the ability to detect such rare events, indicating that our cohort was underpowered for this outcome and no definite conclusion should be drawn regarding the potential influence of elevated PRAETORIAN score and higher shock impedance on DFT success.

Implantation duration was not influenced by obesity in the current study, consistent with the findings of Brouwer et al., who reported similar procedure times in patients with and without obesity [25]. However, higher BMI—as a continuous variable (per 1 kg/m^2^)—was associated with increased procedure time in the UNTOUCHED trial [29]. Notably, in addition to BMI, factors such as the incision technique used, the application of imaging modalities, and the performance of DFT may influence procedure duration as pointed out by Boersma and colleagues [29].

### Long-term outcomes

No difference was observed between patients with and without obesity regarding the risk of appropriate and inappropriate shock delivery in the current study. The long-term risk of appropriate therapies in relation to BMI as a continuous variable was previously assessed in the pooled analysis [23] from the Subcutaneous ICD Post Approval Study [30], and the EFFORTLESS registry [4], and revealed an increasing tendency for appropriate shock delivery in patients with obesity. These results should, however, be interpreted with the understanding that obesity itself may represent an independent risk factor for ventricular arrhythmias [16, 17, 31]. Our results—regarding the equal risk of inappropriate shocks between individuals with and without obesity—are in line with the findings from the early Investigational Drug Exemption (IDE) trial [32], as well as the meta-analysis [23] of the data from the Subcutaneous ICD Post Approval Study [30], the EFFORTLESS registry [4], and the UNTOUCHED trial [33].

Regarding clinical shock efficacy, Frankel et al. previously evaluated the defibrillation efficacy of the first 80 J shock to terminate spontaneous ventricular arrhythmias among three different BMI groups (underweight and normal [BMI < 25 kg/m^2^], overweight [BMI 25–29.9 kg/m^2^] and with obesity [BMI ≥ 30 kgm^2^]). They did not detect difference regarding this parameter; however, higher BMI was associated with an increased risk of “any first shock failing”, including failed shocks during implant and chronic defibrillation testing, as well as failed shock during spontaneous ventricular arrhythmias [32]. In the S-ICD Post Approval Study, lower BMI proved to be a predictor of having a successful first shock [30]. Reassuringly, in the current study, we revealed a 100% success rate in terminating VT/VF by the first clinical shock in both groups, irrespective of BMI, confirming consistent therapeutic efficacy across the spectrum of body weight.

Notably, we observed a numerically (but not statistically) higher risk for non-infectious complications in the group with obesity; on the other hand, device-related infections were only recorded in the group without obesity. Of note, diabetes mellitus was more prevalent among patients with obesity, which might represent a potential confounder for infectious complications. However, in a univariate logistic regression analysis, diabetes was not significantly associated with the occurrence of infections. Due to the low number of infectious events, further multivariable analysis was not feasible, and the results should therefore be regarded as hypothesis generating. The increased risk of system-related complications in patients with higher BMI values is a well-established and consistently reported finding across prior publications, including the above mentioned recent meta-analysis [23]. The study by Gasperetti at el. (also included in the meta-analysis) also reviewed infectious and non-infectious complications separately and assessed higher BMI as a predictor of non-infectious causes (adjusted HR 1.059, 95% CI 1.014–1.105) [34]. The negative finding of our study that non-infectious complications were not significantly higher in the group with obesity has to be interpreted with caution due to the unequal group sizes, and particularly the relatively low number of patients with obesity in our cohort, which markedly limits the statistical power to detect differences in infrequent events such as complications. In contrast, higher BMI did not emerge as an independent predictor for infectious complications in the study by Gasperetti et al. (adjusted HR 1.042, 95% CI 0.974–1.114) [34], which is fully consistent with the distribution of complication types observed in our study.

Finally, we observed no difference between patients with and without obesity regarding all-cause mortality and rehospitalization (neither device-related nor cardiac-related). Notably, in the IDE trial, the survival rates were also similar among the examined groups (underweight and normal vs. overweight vs. with obesity) [32]. Moreover, obesity was not associated with increased mortality in the EFFORTLESS study (unadjusted HR 1.031; 95% CI 0.992–1.072) [4], comparable to our findings.

### Considerations for clinical practice

Our findings confirm that S-ICD therapy is effective and generally safe in patients with obesity. However, a higher rate of non-infectious complications—such as suboptimal lead or generator positioning and dislodgement over time (possibly due to fluctuating weight)—should be anticipated in this population. On the other hand, the S-ICD may represent a valuable option for patients with obesity, as they often require ICD implantation at a younger age [35, 36]. In this context, avoiding transvenous leads and preserving venous access may offer significant long-term benefits, making the S-ICD a strategically favourable choice for this population.

While pre-implant ECG screening is generally unaffected by obesity [23], anatomical factors may still complicate pre-implantation imaging and procedural planning [34]. As recommended by the manufacturer, fluoroscopy may be used to confirm the position of the heart silhouette and to assist in determining appropriate system component placement and incision sites prior to prepping and draping the patient. Skin markings should be made accordingly to guide the implant procedure [37]. In patients with increased subcutaneous adipose tissue, these landmarks may become less reliable once sterile drapes and adhesive film dressings are applied, potentially altering the anatomical orientation. Therefore, in patients with obesity, additional intraoperative fluoroscopic verification—particularly using lateral projections—may be considered routinely to ensure appropriate lead/device positioning and to assess thickness of the subcoil/subgenerator fat. It should be noted, however, that obtaining lateral fluoroscopic views may require additional setup considerations to preserve sterile technique while properly positioning the C-arm.

**Fig. 4:**
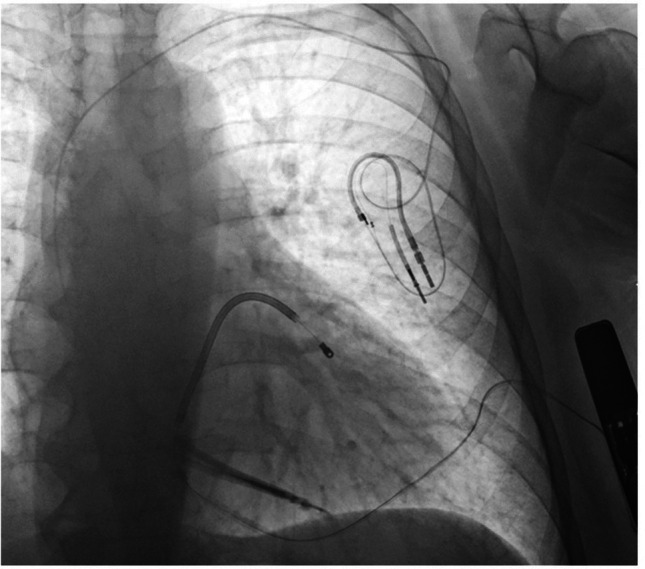
Upright X-ray image of an S-ICD recipient with extreme obesity (BMI 54 kg/m^2^). The 44-year-old female patient with idiopathic VF and a transvenous ICD implanted 14 years earlier underwent S-ICD implantation due to lead fracture. Given the high surgical risk and an occluded left subclavian vein, S-ICD implantation with abandonment of the old lead was preferred over a transvenous lead extraction. Despite successful DFT and appropriate lead positioning confirmed by intraoperative fluoroscopy, the upright chest X-ray obtained the following day revealed displacement of the distal lead tip, likely due to postural shift of excessive subcutaneous fat. The lead was subsequently repositioned using the 3-incision technique, which may have prevented the dislocation initially

Another practical consideration is the choice between the 2-incision and 3-incision implantation techniques. Most centers now prefer the 2-incision method for its simplicity, faster procedural time [24], and improved cosmetic results [38]. In our cohort, implantations were preferably performed using the standardized two-incision approach. Nevertheless, in patients with extreme obesity, the three-incision technique may offer better electrode fixation and reduced risk of lead displacement by allowing direct visualization and anchoring of the parasternal lead (Fig. 4). This consideration is particularly relevant in anatomically challenging cases but warrants prospective validation in larger, dedicated studies.

### Limitations

This was a retrospective analysis, and therefore all the potential limitations of such a study design apply. Since the patient groups were not randomized and were compared based on observational allocation (i.e. BMI at implantation), residual confounding cannot be entirely excluded. Notably, minimal differences were observed in baseline characteristics; however, missing data for certain clinical and echocardiographic variables (e.g., LVEF, LVEDD) may have introduced residual confounding, and subtle unmeasured differences between groups cannot be fully ruled out. Moreover, follow-up data were not complete for all patients, and long-term follow-up of 7–10 years was not uniformly available, which may have led to underreporting of late complications or device-related events. Although this was a bicentric cohort, the number of patients with obesity remained relatively low, limiting the statistical power to detect differences in infrequent outcomes, such as complications and mortality.

Moreover, not all components required to calculate the PRAETORIAN score were systematically recorded, and therefore the score could be provided only for a small subset of patients. Notably, the clinical relevance of the PRAETORIAN score has recently been questioned by a large multicenter study, which suggested that in contemporary practice—especially when using the intermuscular implantation technique—most components of the score become redundant [39]. In the study by Ziacchi et al., BMI and shock impedance emerged as key predictive markers for an elevated PRAETORIAN score, both of which were comprehensively evaluated in our analysis.

## Conclusions

In this bicentric retrospective study, S-ICD therapy proved to be equally effective in patients with and without obesity. Despite a significantly higher intraoperative shock impedance in patients with obesity, DFT success rates and clinical shock efficacy remained high in both groups. Moreover, during long-term follow-up, no significant differences were observed in the rates of appropriate or inappropriate shocks, survival, or cardiac/device-related rehospitalizations.

However, the distinct system-specific complication profile associated with obesity should be highlighted: while infectious complications occurred only in patients without obesity, non-infectious complications were numerically more frequent in the patient group with obesity, consistent with previous reports. These findings support the broader use of S-ICD therapy in patients with obesity, including younger individuals, where long-term preservation of venous access is particularly important. At the same time, our data highlight the need for meticulous preoperative planning—including accurate skin marking, intraoperative fluoroscopic verification, and the involvement of experienced operators—to adapt to challenging anatomical conditions and to help ensure optimal system positioning.

## Data Availability

The data that support the findings of this study are available from the corresponding author [JWE], upon reasonable request.

## Abbreviations

AAD: Antiarrhythmic drugs
ACE: Angiotensin-converting enzyme
ARB: Angiotensin receptor antagonist
ARNI: Angiotensin receptor neprilysin inhibitor
BMI: Body mass index
CCI: Charlson Comorbidity Index
CI: Confidence interval
CKD: Chronic kidney disease
CKM: Cardiovascular-kidney-metabolic syndrome
DFT: Defibrillation testing
DOAC: Direct oral anticoagulants
ECG: Electrocardiogram
EHRA: European Heart Rhythm Association
HR: Hazard ratio
ICD: Implantable cardioverter defibrillator
ICM: Ischemic cardiomyopathy
IQR: Interquartile range
LVEDD: Left ventricular end-diastolic diameter
LVEF: Left ventricular ejection fraction
MI: Myocardial infarction
MRA: Mineralocorticoid receptor antagonist
PAD: Peripheral artery disease
SCD: Sudden cardiac death
S-ICD: Subcutaneous implantable cardioverter defibrillator
SR: Sinus rhythm
VF: Ventricular fibrillation
VKA: Vitamin K antagonists
WHO: World Health Organization

## Code availability

N/A.
